# Incidence and prognosis of thyroid cancer in children: based on the SEER database

**DOI:** 10.1007/s00383-022-05069-3

**Published:** 2022-01-29

**Authors:** Bing Zhang, Wenming Wu, Xiaofei Shang, Deliang Huang, Mingbo Liu, Liang Zong

**Affiliations:** 1 Zhantansi Outpatient, Central Medical District of Chinese, PLA General Hospital, Beijing, 100832 China; 2 College of Otolaryngology Head and Neck Surgery, Chinese PLA General Hospital, No.28 Fuxing Road, Haidian District, Beijing, 100853 China; 3 National Clinical Research Center for Otolaryngologic Diseases, Beijing, 100853 China

**Keywords:** Childhood thyroid cancer, Incidence trend, Join-point regression model, Prognosis factors

## Abstract

**Objective:**

To update the recognition of the trends in the incidence of childhood thyroid cancer (TC) and its prognosis.

**Methods:**

A large-scale sample based on long time-line public database was recruited. Join-point regression model was used to analyze the incidence trend of childhood TC. Univariable and multivariable Cox regression model analyses were applied to explore the survival situation and prognostic factors.

**Results:**

The incidence rate of childhood TC increased between 1975 and 2016 from 3.8/million (95% CI 2.6–5.5) to 11.5/million (95% CI 9.2–14.1), AAPC = 2.38% (95% CI 1.98–9.65) and could be divided into two stages of increasing trends. The incidence rate of Trend1 (1975–2005) increased slowly (APC = 1.08%, 95% CI 0.38–1.82) while Trend2 (2005–2016) increased dramatically (APC = 6.77%, 95% CI 4.30–9.28). Annual incidence rate of small size tumor (< 4 cm) and local stage childhood TC increased significantly. The overall cumulative survival rate for childhood TC was high up to 97–99%. Males, black race, MTC type, distant metastasis, tumor size ≥ 4 cm, non-primary cancer were the independent risk factors of childhood TC prognosis.

**Conclusion:**

A contribution of overdetection to rising pediatric TC rates might not be able to rule out. For clinical implications, screening TC in children with potential specific risk factors is feasible. Over-treatment to small size and local stage TC in children should be avoided.

**Supplementary Information:**

The online version contains supplementary material available at 10.1007/s00383-022-05069-3.

## Introduction

Thyroid cancer (TC) is a common endocrine malignant tumor in both adults and children whose incidence has been increasing steadily for decades in the world [1, 2]. For example, TC incidence increased, on an average of 3.6% per year during 1974–2013 in the USA [3]. With improvements in diagnostic methods and implementation of advanced imaging modalities in the past decades, the diagnosis of micro-TC (< 1 cm) has been promoted, which would hardly cause symptoms or require treatment, explaining a substantial proportion of the increase of TC [3, 4].

Although TC is likely to be over-diagnosed among adults [5, 6], children are less frequent to receive thyroid screening or imaging examinations of the neck [7, 8]. Therefore, studying the incidence trend of childhood TC might better reflect the prevalence of this disease in the population. In addition, some types of childhood TC are more prone to have lymph node metastasis than in adults, with a wider range of lesions and leading a worse prognosis [9, 10]. Currently, many studies have reported the incidence and prevalence of TC in children and adolescents worldwide, with the age ranges from 0 to 20 years [11–13]. Most of the researches focus on exploring risk factors and the survival rate for childhood TC but pay less attentions to the prognosis factors on account of the very low mortality [14–18].

This study intended to update the recognition of the trends in the incidence of childhood TC and the prognosis factors through a large and public population-based cohort that are more time-span. First, the incidence of childhood TC was fitted using the join-point regression (JPR) model to explore its changing trend and its distribution in the population. Then, univariable and multivariable Cox regression model analyses were applied to explore the survival situation and prognostic factors of childhood TC. From epidemiological respect, we hope our study could provide more comprehensive acknowledgment for the prevalence and distribution of childhood TC. For clinical implications, we intended to provide constructive advice for clinical decision of TC in children.

## Materials and methods

### Data resource

Data in this study were attained from the Surveillance, Epidemiology, and End Results (SEER) program, one of the most representative databases of large oncology registries worldwide maintained by the United States government and its National Cancer Institute [19]. As an ongoing longitudinal program proceeding for decades, the SEER research reports annual national cancer statistics (approximately 10% of the United States cancer population) on incidence and survival and provides population data associated by age, sex, race, year of diagnosis, geographic areas, etc. on its website (https://seer.cancer.gov/data-software/), serving as a platform for offering systematic evidence to studies of cancer-related health disparities [20]. Information of childhood TC with age ranges from 0 to 19 years downloaded from “Incidence-SEER 18 Regs Custom Data (with additional treatment fields), Nov 2018 Sub (1975–2016)”.

TC patients were classified into histology subtypes based on the histology codes in the International Classification of Disease for Oncology, 3rd edition (ICD-O-3) SEER site/histology validation list 2015 [21], including differentiated TC (DTC) and undifferentiated TC (UTC). The histology subtypes are specifically identified using the following ICD-0-3 codes: Classic Papillary Thyroid Cancer (C-PTC): 8050/3, 8260/3, and 8343/3; Variant Papillary Thyroid Cancer (V-PTC): 8340/3, 8350/3, 8344/3, 8052/3, 8130/3, and 8342/3; FTC (Follicular Thyroid Cancer): 8330/3, 8331/3, 8332/3, and 8335/3; MTC (Medullary Thyroid Cancer): 8345/3, 8510/3, 8346/3, and 8347/3; and ATC (Anaplastic Thyroid Cancer): 8021/3 [21]. C-PTC, V-PTC, and FTC were classified as DTC while MTC and ATC were recognized as UTC. In this study, we collected types of C-PTC, V-PTC, FTC and MTC.

For cancer-directed surgery status, the “Subtotal or Near Total Thyroidectomy” and “Total Thyroidectomy” groups were combined to form “Total Thyroidectomy” group. The “Lobectomy” group consisted of TC patients who underwent lobectomy, with or without subsequent isthmusectomy. If a patient initially underwent a thyroid lobectomy and then went on to have a completion thyroidectomy, his/her surgery status in SEER was coded as “Total Thyroidectomy” [22].

In this study, we used the software SEER*Stat (version 8.3.9) to retrieve the complete TC cases with an age under 20 and a time span from 1975 to 2016 in the SEER database. A total of 4343 eligible patients were finally extracted according to pathologically confirmed diagnosis and integrated survival information. And annual standardized TC incidence rates were obtained. The following demographic and clinicopathological variables were included in our analysis: age, sex, race, histologic type, metastasis, radiation, chemotherapy, grade, surgery, AJCC stage, TNM stage, tumor size, primary cancer and survival information. Age groups were divided by 5 years per group. Due to the small number of patients aged 0–9 years, they were combined into one group. Race was aggregated into White, Black and others (American Indian/Alaskan Native or Asian/Pacific Islander). The primary outcomes in our study were overall survival (OS). OS was calculated as the time from diagnosis to death of TC.

### Join-point regression model

The traditional regression analysis mainly reflects the overall trend of global data, but may not reveal the specific trend of local data. Therefore, the piecewise regression model comes into being [23]. The basic idea of the JPR model proposed by Kim et al. is to divide a long-term trend line into several statistically significant trend segments by model fitting, and each segment is described by continuous linearity, which could be linear data model or log-linear data model [24, 25]. Based on the annual incidence, this model mainly provides two outcome indicators: annual percent change (APC) and average annual percent change (AAPC) and corresponding confidence intervals (CIs). As a trend analysis of time series data, JPR model has been widely used in the field of epidemiological trends of cancer and chronic diseases, mainly to analyze the characteristics of temporal trends of morbidity and mortality [26–28].

### Statistical methods

Summary statistics was used to describe the demographic characteristics and clinical data at baseline. The measurement data of normal distribution were described by means ± SD; the non-normal data were described by median and quartile range [*M* (Q1, Q3)]. Enumeration data were described by number of cases and composition ratio [*N*(%)]. Incidence rates were calculated per 100,000 persons and age-adjusted to the 2000 US Standard Population using SEER*Stat (version 8.3.9). The JPR model analysis was performed using Join-point Regression Program4.8 and trend curves were presented using three-year rolling average for each calendar year. Survival analysis was performed with Cox proportional hazard regression model using R package survival*.* Kaplan–Meier (K–M) curve was used to show the survival curves. Log-rank test evaluated the statistically significant differences of survival. All other statistical analyses were performed using R4.0.2. Bilateral tests were used for all statistical tests. A value of *P* < 0.05 was considered statistically significant.

## Results

### Demographics and incidence of childhood TC

#### Demographics of childhood TC

A total of 4343 TC cases under 20 years old were included. Age 0–9 group consisted of 213 cases, accounting for 4.90% of the overall subjects; age 10–14 group consisted of 904 cases (20.82%); age 15–19 group consisted of 3,226 cases (74.28%). Male subjects were 824 cases accounted for 18.97% while female subjects were 3,519 cases accounted for 81.03%. Black race were 233 cases (5.23%), white race were 3,602 cases (84.44%), and other race were 441 cases (10.34%). The median follow-up time was 104.00 (Q1: 46.00, Q3: 193.00) months. The characteristics of demographics and clinic information at baseline are displayed in Table 1.

**Table 1:** Characteristics of demographics and clinical information of childhood TC

Variables	Total (*n* = 4343)	Alive (*n* = 4229)	Dead (*n* = 114)
Age, *n* (%)			
00–09 years	213 (4.90)	205 (4.85)	8 (7.02)
10–14 years	904 (20.82)	877 (20.74)	27 (23.68)
15–19 years	3226 (74.28)	3147 (74.41)	79 (69.30)
Sex, *n* (%)			
Female	3519 (81.03)	3448 (81.53)	71 (62.28)
Male	824 (18.97)	781 (18.47)	43 (37.72)
Race, *n* (%)			
Black	223 (5.23)	208 (5.01)	15 (13.16)
White	3602 (84.44)	3517 (84.71)	85 (74.56)
Other	441 (10.34)	427 (10.28)	14 (12.28)
Histologic type, *n* (%)			
C-PTC	2660 (63.32)	2601 (63.52)	59 (55.66)
V-PTC	1002 (23.85)	981 (23.96)	21 (19.81)
FTC	352 (8.38)	344 (8.40)	8 (7.55)
MTC	187 (4.45)	169 (4.13)	18 (16.98)
Metastasis, *n* (%)			
Distant	217 (5.12)	194 (4.69)	23 (21.30)
Localized	2062 (48.61)	2018 (48.81)	44 (40.74)
Regional	1963 (46.28)	1922 (46.49)	41 (37.96)
Radiation, *n* (%)			
No	1982 (45.64)	1923 (45.47)	59 (51.75)
Yes	2361 (54.36)	2306 (54.53)	55 (48.25)
Chemotherapy, *n* (%)			
No	4324 (99.56)	4214 (99.65)	110 (96.49)
Yes	19 (0.44)	15 (0.35)	4 (3.51)
Grade, *n* (%)			
I	769 (17.71)	754 (17.83)	15 (13.16)
II	169 (3.89)	167 (3.95)	2 (1.75)
III	50 (1.15)	42 (0.99)	8 (7.02)
IV	10 (0.23)	8 (0.19)	2 (1.75)
Unknown	3345 (77.02)	3258 (77.04)	87 (76.32)
Surgery, *n* (%)			
No surgery	59 (1.36)	56 (1.32)	3 (2.63)
Lobectomy	342 (7.87)	339 (8.02)	3 (2.63)
Total thyroidectomy	3032 (69.81)	2988 (70.66)	44 (38.60)
Unknown	910 (20.95)	846 (20.00)	64 (56.14)
AJCC stage, *n* (%)			
I	2337 (53.81)	2316 (54.76)	21 (18.42)
II	70 (1.61)	69 (1.63)	1 (0.88)
III and IV	25 (0.58)	23 (0.54)	2 (1.75)
Unknown	1911 (44.00)	1821 (43.06)	90 (78.95)
T stage, *n* (%)			
T1	998 (22.98)	990 (23.41)	8 (7.02)
T2	595 (13.70)	589 (13.93)	6 (5.26)
T3	699 (16.09)	695 (16.43)	4 (3.51)
T4	97 (2.23)	94 (2.22)	3 (2.63)
Unknown	1954 (44.99)	1861 (44.01)	93 (81.58)
N stage, *n* (%)			
N0	1292 (29.75)	1278 (30.22)	14 (12.28)
N1	1151 (26.50)	1138 (26.91)	13 (11.40)
Unknown	1900 (43.75)	1813 (42.87)	87 (76.32)
M stage, *n* (%)			
M0	2372 (54.62)	2349 (55.55)	23 (20.18)
M1	67 (1.54)	66 (1.56)	1 (0.88)
Unknown	1904 (43.84)	1814 (42.89)	90 (78.95)
Tumor size (cm), *n* (%)			
< 1	599 (13.79)	591 (13.97)	8 (7.02)
1–	972 (22.38)	959 (22.68)	13 (11.40)
2–	1423 (32.77)	1401 (33.13)	22 (19.30)
≥ 4	726 (16.72)	707 (16.72)	19 (16.67)
Unknown	623 (14.34)	571 (13.50)	52 (45.61)
Primary cancer, *n* (%)			
No	123 (2.83)	109 (2.58)	14 (12.28)
Yes	4220 (97.17)	4120 (97.42)	100 (87.72)
Survival months, *M* (*Q*_1_,*Q*_3_)	104.0 (46.0,193.0)	104.0 (46.0,192.0)	97.5 (36.0,264.0)

Some data are missing

#### Overall incidence of childhood TC

**Fig. 1:**
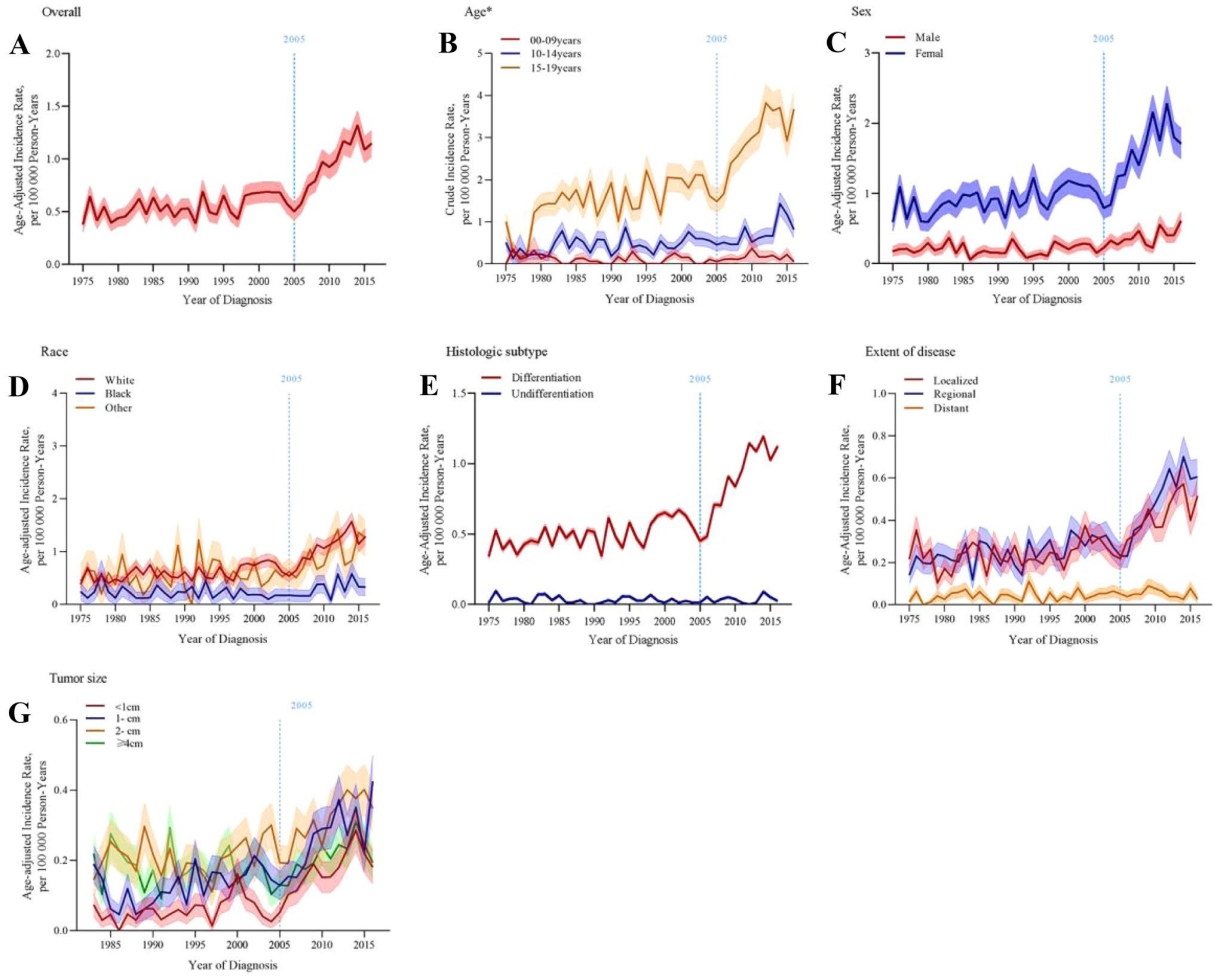
Distribution of childhood TC incidence trend chart (**A** Overall, **B** Age, **C** Sex, **D** Race, **E** Histologic subtype, **F** Extent of disease, **G** Tumor size)

Table 2 and Fig. 1 show the JPR analysis of trends in incidence. From 1975 to 2016, the overall incidence of childhood TC increased steadily, with age-standardized incidence rate (ASR) from 3.8/million (95% CI 2.6–5.5) to 11.5/million (95% CI 9.2–14.1), AAPC = 2.38% (95% CI 1.98–9.65). The incidence could be divided into two stages of trends. Trend1: between 1975 and 2005, the ASR of childhood TC increased slowly with APC = 1.08% (95% CI 0.38–1.82, *P* < 0.05). Trend2: the ASR increased significantly from 2005 to 2016 with APC = 6.77% (95% CI 4.30–9.28, *P* < 0.001). See Fig. 1A.

**Table 2:** JPR analysis of trends in incidence of childhood TC

Incidence	Trend 1		Trend 2		AAPC
	Years	APC % (95% CI)	Years	APC % (95% CI)	1975–2016
Overall	1975–2005	1.08 (0.38–1.82)*	2005–2016	6.77 (4.30–9.28)*	2.38 (1.98–9.65)*
Age^b^					
00–09 years	NA	NA	NA	NA	NA
10–14 years	1975–2016	2.25 (1.18–3.32)*			2.25 (1.18–3.32)*
15–19 years	1975–2005	1.83 (0.82–2.92) *	2005–2016	6.28 (3.02–9.65)*	2.98 (2.41–3.61)*
Sex					
Female	1975–2005	1.19 (0.41–2.00)*	2005–2016	6.57 (3.63–9.67)*	2.43 (1.90–3.00)*
Male	1975–2016	2.19 (1.29–3.11)*			2.19 (1.29–3.11)*
Race					
White	1975–2005	1.37 (0.62–2.24)*	2005–2016	6.70 (3.68–9.76)*	2.57 (2.01–3.12)*
Black	1975–2016	0.86 (− 0.42–2.12)			0.86 (− 0.42–2.12)
Other	NA	NA	NA	NA	NA
Tumor size					
< 1	NA	NA		NA	NA
1–	1983–2016	4.09 (2.89–5.31)*			4.09 (2.89–5.31)*
2–	1983–2016	2.22 (1.38–3.01)*			2.22 (1.38–3.01)*
≥ 4	1983–2005	− 1.1 (− 2.89–0.71)	2005–2016	4.78 (0.10–9.91)*	0.58 (− 0.38–1.52)
Metastasis					
Localized	1975–2005	0.57 (− 0.33–1.63)	2005–2016	5.00 (1.74–8.36)*	2.13 (1.45–2.78)*
Regional	1975–2005	1.45 (0.56–2.34)*	2005–2016	7.95 (4.89–11.13)*	3.03 (2.38–3.67)*
Distant	1975–2016	1.16 (− 2.13–4.56)			1.16 (− 2.13–4.56)
Histologic type					
Differentiated	1975–2005	1.27 (0.61–1.92)*	2005–2016	4.75 (1.78–7.82)*	3.01 (2.52–3.54)*
Undifferentiated	1975–2016	4.52 (− 12.10–24.31)			4.52 (− 12.10–24.31)

*AAPC* average annual percent change, *APC* annual percent change, *NA* not applicable (no sufficient data for analysis)

*The APC or AAPC is significantly different from zero (*P* < 0.05)

#### Distribution of incidence of childhood TC

We performed subgroup analyses of incidence by some interested population groups. Due to lack of data, the results of “0–9 years” group in “Age”, “Other” group in “Race” and “ < 1 cm” group in “Tumor size” were not applicable. From Table2 and Fig. 1, the incidence of “10–14 years” group had no “join-point” and increased gradually from 1975 to 2016 (*P* < 0.05) and so as “Male” group and “1–4 cm” (*P* < 0.05). Differently, note that the incidence of “15–19 years” group, “Female” group, “White” group, “Differentiated” group and “Regional” group increased gradually from 1975 to 2005 (*P* < 0.05) but sharply increased after 2005 (*P* < 0.05), just as the tendency of overall incidence. Incidence trend of “Localized” group also suddenly increased after 2005 (*P* < 0.05). Besides, the incidence of “Black”, “Distant” and “Undifferentiated” had no significantly increase (*P* > 0.05).

### Risk factors for the prognosis of childhood TC

#### Univariable risk factors analysis

Table 3 showed results of single risk factors analysis of prognosis in childhood TC. As shown in Table 3, there were no significant differences in risk of death between age groups. Male patients had 2.595 times of death risk compared to females (hazard ratio, HR 2.595, 95% CI 1.776–3.790). The black race patients had 3.029 times of death risk compared to the white race (HR 3.029, 95% CI 1.749–5.245).

**Table 3:** Result of single risk factors analysis of prognosis of childhood TC

Variables	*β*	SE	*χ*^2^	*P*	HR	95% CI	
						Lower	Upper
Age							
00–09 years					Ref		
10–14 years	− 0.169	0.403	0.177	0.674	0.844	0.383	1.859
15–19 years	− 0.398	0.371	1.150	0.284	0.672	0.325	1.390
Sex							
Female					Ref		
Male	0.953	0.193	24.320	< 0.001	2.595	1.776	3.790
Race							
White					Ref		
Black	1.108	0.280	15.645	< 0.001	3.029	1.749	5.245
Other	0.431	0.289	2.228	0.136	1.539	0.874	2.712
Histologic type							
MTC					Ref		
C-PTC	− 1.263	0.270	21.844	< 0.001	0.283	0.166	0.480
V-PTC	− 1.365	0.322	17.990	< 0.001	0.255	0.136	0.480
FTC	− 1.448	0.425	11.598	< 0.001	0.235	0.102	0.541
Metastasis							
Localized					Ref		
Regional	− 0.011	0.217	0.002	0.961	0.989	0.647	1.514
Distant	1.437	0.258	31.109	< 0.001	4.209	2.540	6.975
Radiotherapy							
Yes					Ref		
No	0.143	0.194	0.543	0.461	1.153	0.789	1.686
Chemotherapy^a^							
Yes					Ref		
No	− 1.989	0.511	15.153	< 0.001	0.137	0.050	0.373
Grade^b^							
I					Ref		
II	− 0.498	0.754	0.436	0.509	0.608	0.139	2.663
III	2.137	0.440	23.631	< 0.001	8.471	3.579	20.047
IV	2.519	0.760	10.999	< 0.001	12.418	2.802	55.033
Surgery							
No surgery					Ref		
Lobectomy	− 1.825	0.817	4.993	0.025	0.161	0.033	0.799
Total thyroidectomy	− 1.277	0.597	4.575	0.032	0.279	0.087	0.899
AJCC stage^b^							
I					Ref		
II	0.423	1.024	0.170	0.680	1.526	0.205	11.349
III and IV	2.333	0.745	9.793	0.002	10.305	2.391	44.413
T stage^b^							
T1					Ref		
T2	0.177	0.540	0.107	0.743	1.194	0.414	3.441
T3	− 0.319	0.612	0.272	0.602	0.727	0.219	2.413
T4	1.263	0.677	3.479	0.062	3.536	0.938	13.332
N stage^b^							
N0					Ref		
N1	0.082	0.385	0.045	0.832	1.085	0.510	2.310
M stage^b^							
M0					Ref		
M1	0.383	1.022	0.141	0.708	1.467	0.198	10.864
Tumor size							
< 1					Ref		
1–	− 0.100	0.450	0.050	0.823	0.905	0.375	2.183
2–	− 0.070	0.414	0.028	0.866	0.933	0.415	2.098
≥ 4	0.446	0.423	1.112	0.292	1.562	0.682	3.581
Primary cancer							
No					Ref		
Yes	− 1.684	0.286	34.762	< 0.001	0.186	0.106	0.325

^a^The result had bias

^b^A large amount of missing data

In terms of clinic factors, due to mass of missing data (44–77%, see Table 1) of “grade”, “AJCC stage” and “TNM stage”, we are cautious of these results. And because very few subjects (19 cases, 0.44%) received chemotherapy, its result had bias. In “Histologic type” group, compared to “MTC”, other histologic types had lower death risk (*P* < 0.001). The distant metastasis patients had much higher death risk compared to localized patients (HR 4.209, 95% CI 2.540–6.975). Patients who received lobectomy (HR 0.161, 95% CI 0.033–0.799) and total thyroidectomy (HR 0.279, 95% CI 0.087–0.899) had a reduced risk of death compared with those who did not undergo surgery. Patients who were “Primary cancer” had much lower death risk compared to those who were not (HR 0.186, 95% CI 0.106–0.325). Besides, whether received radiotherapy or not and tumor size had no significant difference for death risk in univariable analysis (*P* > 0.05).

#### Multivariate risk factors analysis

Variables that were statistically significant in the univariable analysis, such as sex, race, histologic type, surgery type, metastasis and primary cancer, and common influencing factors like age and tumor size [21], were included in the multiple factors Cox regression model analysis. Finally, factors that were included in the regression model are presented in Table 4. Males (HR 2.659, 95% CI 1.524–4.641), black race (HR 2.345, 95% CI 1.027–5.355), MTC type (C-PTC, HR 0.160, 95% CI 0.072–0.355; FTC, HR 0.185, 95% CI 0.058–0.586; V-PTC, HR 0.127, 95% CI 0.050–0.323), distant metastasis (HR 2.884, 95% CI 1.296–6.419), tumor size ≥ 4 cm (HR 3.071, 95% CI 1.141–8.264), non-primary cancer (primary cancer, HR 0.089, 95% CI 0.043–0.185) were independent risk factors of the prognosis.

**Table 4:** Result of multiple risk factors analysis of prognosis of childhood TC

Variables	*β*	SE	*χ*^2^	*P*	HR	95% CI	
						Lower	Upper
Sex							
Female					Ref		
Male	0.978	0.284	11.855	< 0.001	2.659	1.524	4.641
Race							
White					Ref		
Black	0.852	0.421	4.096	0.043	2.345	1.027	5.355
Other	− 0.176	0.480	0.134	0.714	0.839	0.327	2.149
Histologic type							
MTC					Ref		
C-PTC	− 1.832	0.406	20.377	< 0.001	0.160	0.072	0.355
V-PTC	− 2.064	0.476	18.787	< 0.001	0.127	0.050	0.323
FTC	− 1.690	0.590	8.209	0.004	0.185	0.058	0.586
Metastasis							
Localized					Ref		
Regional	0.030	0.324	0.008	0.927	1.030	0.546	1.944
Distant	1.059	0.408	6.736	0.009	2.884	1.296	6.419
Tumor size							
< 1					Ref		
1–	0.604	0.517	1.367	0.242	1.829	0.665	5.034
2–	0.731	0.486	2.261	0.133	2.076	0.801	5.381
≥ 4	1.122	0.505	4.933	0.026	3.071	1.141	8.264
Primary cancer							
No					Ref		
Yes	− 2.421	0.375	41.716	< 0.001	0.089	0.043	0.185

#### K–M survival curves analysis

**Fig. 2:**
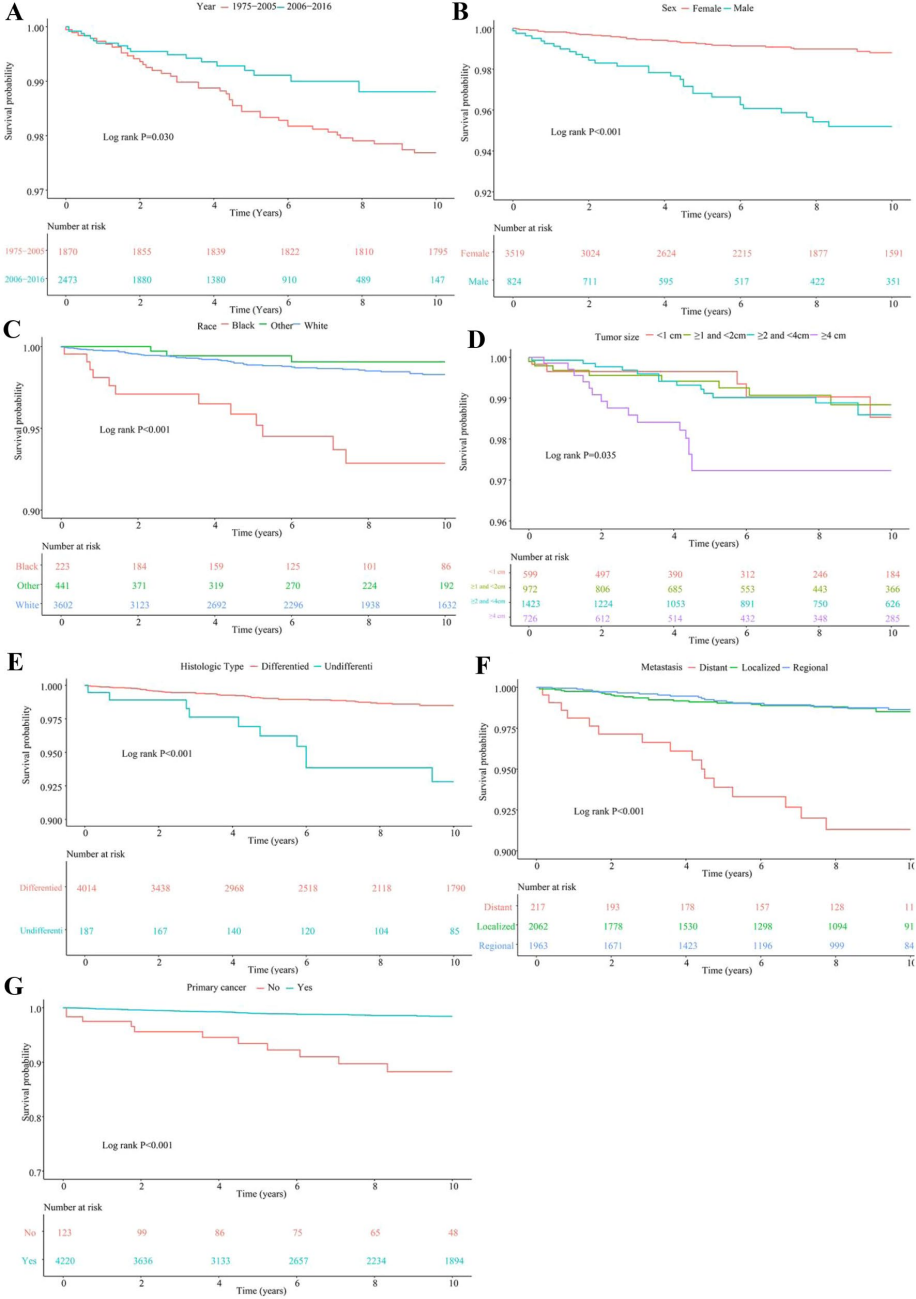
Factors affecting the grouping of KM curve (**A** Year, **B** Sex, **C** Race, **D** Tumor size, **E** Histologic subtype, **F** Extent of disease, **G** Primary lung cancer)

K–M survival curves for the above independent risk factors as well as the overall survival are shown in Fig. 2, Log-rank test, see in Table S1. The overall cumulative survival rate for childhood TC from 1975 to 2005 was 97.70% and with a promotion to 99.27% from 2006 to 2016 (*P* = 0.030). From 1975 to 2016, the survival rate for female was 97.98% and higher than that of male with 94.78% (*P* < 0.001). The cumulative survival rate for race of white, black and other was 97.64%, 93.27% and 96.83%, respectively, and had a difference (*P* < 0.001). The cumulative survival rate for DTC was up to 98.88% and higher than that of UTC with 94.65% (*P* < 0.001). The cumulative survival rate for tumor size < 4 cm was around 99.15% and higher than that of size ≥ 4 cm with 97.80% (*P* = 0.035). Cumulative survival rate for localized, regional and distant metastasis was 97.78%, 97.91% and 89.40% with a significant difference (*P* < 0.001). The cumulative survival rate for primary cancer was 97.63% and much higher than that of non-primary cancer with 88.62% (*P* < 0.001).

## Discussion

The pattern of TC incidence in children and adolescents is similar to that in adults [29]. Compared to frequent over-diagnoses in TC among adults, children and adolescents are less likely to have overdetection effects from a clinical perspective [8, 11, 20, 30, 31]. Therefore, studying the incidence trend and exploring the prognostic factors of childhood TC might better reflect the prevalence of this disease and offer useful clinical treatment suggestions. This study analyzed the trends in the incidence of childhood TC with distribution and explored the prognosis factors using JPR model and survival analysis, based on large samples with longitudinal data from the SEER database between 1975 and 2016.

The overall ASR of childhood TC increased from 3.8/million (95% CI 2.6–5.5) to 11.5/million (95% CI 9.2–14.1) between 1975 and 2016, with AAPC = 2.38% (95% CI 1.98–9.65) and could be divided into two stages of increasing trends. Between 1975 and 2005 (Trend1), the incidence of childhood TC increased slowly (APC = 1.08%, 95% CI 0.38–1.82) while it had increased significantly (APC = 6.77%, 95% CI 4.30–9.28) since 2005 (Trend2). In the same time period, the overall incidence of childhood TC in Denmark increased significantly from 3.6/million to 9.7/million between 1980 and 2014, with an AAPC of 2.9% [15]. The growth trends of these two countries were roughly the same. Trend1 was lower than incidence trend of childhood TC in Europe of 3% per year during 1978–1997 [14]. Trend2 was consistent with the study [18] (APC = 5.90% during 2007–2012). A national study [11] in the USA only reported one trend that pediatric DTC incidence rates increased from 4.77/million (95% CI 4.26, 5.33) to 8.82/million (95% CI 8.13, 9.56) between 1988 and 2013. This incidence trend was similar to that of the present study during the same period (see Fig. 1A). Nevertheless, the advantage of this study is that we used a much longer timeline to observe the incidence trends of childhood TC, so that a fast-growing period was found (Trend2, since 2005).

The incidence of TC is known to vary widely according to age, gender and race/ethnicity. Low in the first years of age, the incidence rates increase dramatically in adolescence, and are higher in girls than boys [2, 15, 18, 24]. This study is consistent with prior findings. In the analysis of distribution of incidence (Fig. 1), we found that the trends reported for overall incidence were largely driven by “15–19 years” group, “Female” group, “White” group, “Localized” group, “Regional” group and DTC types (papillary TC, 87%). Physiologically, this can be explained by differences in developmental status, hormone levels and race [2, 28, 32]. Besides, we observed that the annual increase rates of distant TC (1.16%) are lower than those observed for localized (2.13%) or regionally (3.03%), which was consistent with the findings of a previous study (2.4%, 4.6%, 4.3%, respectively) in adults TC [3].

Notably, the significant annual increases in the rates of small size tumor (< 4 cm) and local or regional stage TC in children since 2005 (Trend2) suggests that we might not be able to rule out a contribution of early detection (improvements in diagnostic methods) to rising pediatric DTC rates. A global study shows that there is over-diagnosis in childhood TC, too [29]. And rapid increases in TC incidence between 2008 and 2012 were observed in almost all surveyed countries [29]. On the one hand, considering over-diagnosis can lead to over-treatment, lifelong medical care, and side effects, the authors appeal to explicitly recommend against screening for TC in the asymptomatic children and adolescents who are free from specific risk factors [29]. On the other hand, because patients undergoing detection are younger and healthier and are diagnosed with lower-risk TC, they are less likely to die [33]. In particular, it involves children's psychological problems; thus, early diagnosis, active treatment and enhanced follow-up are much required. In summary, we suggest that for children with potential specific risk factors (such as adolescence girls), screening is feasible. Other asymptomatic children should be avoided to over-diagnosis.

We then explored survival and prognosis factors of childhood TC. Generally, the overall cumulative survival rate for childhood TC was high, of 97.70% from 1975 to 2005 and with a promotion to 99.27% from 2006 to 2016 (*χ*^2^ = 4.733, *P* = 0.030). More early diagnosis might have contributed to the improved survival. Female, white race, DTC, all sizes of tumor, localized, regional and primary cancer had a high cumulative survival rate above 97% while the cumulative survival rate of distant metastasis and non-primary cancer was lower than 90% (consistent with [18]) (Table S1). The overall high survival rate for childhood TC was consistent with previous studies [14–17]. Males, black race, distant metastasis, MTC subtype, tumors size ≥ 4 cm and non-primary cancer were the independent risk factors of prognosis in childhood TC. A study of analysis of prognosis in geriatric DTC showed that male, African American, tumors sized over than 4 cm, lymph node metastasis and distant metastasis, were strong risk factors in elderly DTC patients prognosis [34]. Prognostic factors of TC for children were similar to those for the elderly.

Current guidelines recommend total thyroidectomy and radio-ablation for most papillary thyroid cancer in children, which have been criticized as aggressive [35]. In this study, patients with lobectomy (HR 0.161, 95% CI 0.033–0.799) or total thyroidectomy (HR 0.279, 95% CI 0.087–0.899) had similar benefit in reduction of death in the univariable analysis. And radiotherapy had no impact for survival benefit. Similar result was reported in the study of [34]. No difference was found between lobectomy and total thyroidectomy with respect to DSS, and radiation therapy also conferred no apparent advantage. A study about total thyroidectomy vs. thyroid lobectomy for localized papillary TC in children also showed that both two methods had a good prognosis for children [35].

This study has its merits. First, this study is based on a large sample of public database which can provide reliable and powerful evidence. Many of the conclusions in this paper support those in the existing literature. Second, we used JPR model to analyze a long timeline database to observe the incidence trend of TC in children more comprehensively so that a fast-growing period was found. Some limitations need to be considered. First, the inherent limitations of the SEER dataset, such as the typical biases associated with a retrospective analysis and possible coding errors. Second, due to insufficient baseline information of the public database, we included limited potential risk factors into prognosis analysis. More additional potential factors including demographic characteristics, life behavior pattern, and clinical treatment information need to be considered in further researches.

## Conclusion

Analysis based on SEER database revealed that the incidence rate of childhood TC increased between 1975 and 2016 and could be divided into two stages of increasing trends. The incidence rate of Trend1 (1975–2005) increased slowly but Trend2 (2005–2016) increased dramatically. A contribution of overdetection to rising pediatric TC rates might not be ruled out. The overall cumulative survival rate for childhood TC was high. Several risk factors of prognosis of childhood TC were identified. For clinical implications, screening TC in children with potential specific risk factors is feasible. Over-treatment to small size and local stage TC in children should be avoided. Still, additional potential prognosis factors including life behavior pattern, demographic characteristics and clinical treatment information need to be considered in further researches.

## Supplementary Information

Below is the link to the electronic supplementary material.

Supplementary file1 (DOCX 14 kb)

## Data Availability

The datasets generated and/or analyzed during the current study are available in the [SEER] repository, [https://seer.cancer.gov/data-software/].
