# Supplementary material for: Incidence and prognosis of thyroid cancer in children: based on the SEER database
Source: Pediatr Surg Int. 2022 Jan 29;38(3):445–56. doi: 10.1007/s00383-022-05069-3 (PMC8831364; doi:10.1007/s00383-022-05069-3)
Supplement: Supplementary file 1 — Supplementary file1 (DOCX 14 kb) [file 383_2022_5069_MOESM1_ESM.docx]

**TableS1.** Result of multiple risk factors analysis of prognosis of childhood TC

| Variables | OS (10years) | | | |
| --- | --- | --- | --- | --- |
|  | n (%) | Survive (%) | Log-rank | *P* |
| Year |  |  | 4.733 | 0.030 |
| 1975-2005 | 1870 (43.06) | 97.70 |  |  |
| 2006-2016 | 2473 (56.94) | 99.27 |  |  |
| Sex |  |  | 26.218 | <0.001 |
| Female | 3519 (81.03) | 97.98 |  |  |
| Male | 824 (18.97) | 94.78 |  |  |
| Race |  |  | 18.038 | <0.001 |
| White | 223 (5.23) | 97.64 |  |  |
| Black | 3602 (84.44) | 93.27 |  |  |
| other | 441 (10.34) | 96.83 |  |  |
| Histologic type |  |  | 23.689 | <0.001 |
| Differentied | 4014 (95.55) | 98.88 |  |  |
| Undifferentied | 187 (4.45) | 94.65 |  |  |
| Tumor size |  |  | 8.581 | 0.035 |
| <1 | 599 (16.10) | 99.17 |  |  |
| 1- | 972 (26.13) | 99.18 |  |  |
| 2- | 1423 (38.25) | 99.02 |  |  |
| ≥4 | 726 (19.52) | 97.80 |  |  |
| Metastasis |  |  | 44.519 | <0.001 |
| Localized | 2062 (48.61) | 97.87 |  |  |
| Regional | 1963 (46.28) | 97.91 |  |  |
| Distant | 217 (5.12) | 89.40 |  |  |
| Primary cancer |  |  | 43.768 | <0.001 |
| No | 123 (2.83) | 88.62 |  |  |
| Yes | 4220 (97.17) | 97.63 |  |  |
